# Severe Hemolytic Anemia Associated with Mild Pneumonia Caused by *Mycoplasma pneumonia*

**DOI:** 10.1155/2012/649850

**Published:** 2012-09-24

**Authors:** Zafer Kurugol, Serife Sebnem Onen, Guldane Koturoglu

**Affiliations:** Department of pediatrics, Ege University Faculty of Medicine, Izmir, 35100 Bornova, Turkey

## Abstract

We report a case of *M. pneumoniae* infection presenting with severe hemolytic anemia in a 4-year-old girl, with a ten-day history of paleness, weakness, and nonproductive cough. She was very pale and tachycardic. However, she was not tachypneic. Chest examination showed normal breath sounds. No rhoncus or whistling was heard. As the erythrocyte sedimentation rate was excessively elevated, the differential diagnosis primarily comprised hematological malignancies. Direct Coombs' test was positive. Diagnosis of *M. pneumoniae* infection was confirmed by elevated levels of *M. pneumoniae* IgG and IgM antibodies and a chest X-ray suggestive of atypical pneumonia. The patient was treated with clarithromycin and packed red cell transfusion and showed a favorable recovery within ten days after admission. In conclusion, this case demonstrates that severe hemolytic anemia caused by *M. pneumoniae* is not always associated with severe pulmonary involvement, even when the respiratory infection is very mild, *M. pneumoniae* may be the cause of severe anemia.

## 1. Introduction

*Mycoplasma pneumoniae* is a well-recognized cause of upper and lower respiratory tract infections and is usually diagnosed in middle and high school students, as discussed by Clyde [1]. Symptoms and signs caused by *M. pneumonia* infection can be divided into two groups: those caused by respiratory tract infections and those caused by extrapulmonary disease. The most frequent clinical presentation of *M. pneumoniae* infection is tracheobronchitis and pneumonia. Extrapulmonary manifestations associated with *M. pneumonia* include hematologic, dermatologic, neurologic, musculoskeletal, renal, cardiac, and gastrointestinal complications. These extrapulmonary manifestations are uncommon and may present before, during, or after pulmonary signs, as discussed by Waites and Talkington [2]. Hematological complications associated with *M. pneumonia* include hemolytic anemia, thrombocytopenia, thrombotic thrombocytopenic purpura, and hemophagocytosis as discussed by Waltes et al. [3]. We report a case of severe hemolytic anemia associated with *M. pneumoniae* infection in a 4-year-old girl. In this reporting case, severe hemolytic anemia developed in the absence of marked clinical evidence of pneumonia.

## 2. Case

A 4-year-old previously healthy girl was admitted to our hospital for severe anemia. Ten days before her admission, she had developed nonproductive cough and weakness and paleness. No family history of hemolytic attacks was noted. The patient had taken amoxicillin-clavulnate for seven days, but her complaints remained. On physical examination, the patient was very pale and tachycardic (140 beats/min). Blood pressure was 90/60 mmHg and body temperature was 36.8°C. Chest examination showed normal breath sounds. No rhoncus or whistling was heard. Respiratory rate was 20/minute. Systolic flow murmur was heard at every auscultation focus. The rest of the examination was unremarkable.

**Figure 1:**
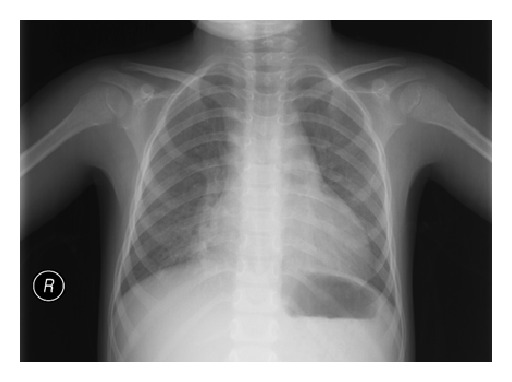
Chest X-ray demonstrating interstitial infiltration.

Admission blood count showed a haemoglobin level of 6.3 g/dl, a mean corpuscular volume of 83.1 fL, and white blood cell count of 11200/mm^3^, with 41% neutrophils, 51% lymphocytes, 8% monocytes, and a platelet count of 569000/mm^3^. The reticulocyte count was 6%. Lactate dehydrogenase (LDH) was 581 U/L (reference range: 100–250 U/L). Total bilirubin was 1.5 mg/dL (normal 0.1–1.0 mg/dL), of which 1.2 mg/dL was indirect (normal 0–0.8 mg/dL). Haptoglobin was 18 mg/dL (normal 30–200 mg/dL). The serum level of C-reactive protein was 0.81 mg/dL (reference range: 0–0.8 mg/dL), and the erythrocyte sedimentation rate exceeded 140 mm in 1 h. Other blood chemistry, liver profile, and coagulation studies were within normal limits. Blood, sputum and urine cultures were negative. The chest X-ray showed bilateral interstitial infiltration (Figure 1).

A bone marrow aspiration was performed to rule out the possibility of an underlying hematological malignancy, and it revealed mildly increased erythropoiesis but no changes indicative of a hematological malignancy were observed. Direct Coombs test was positive. Mycoplasma pneumoniae IgM antibody was positive; *M. pneumoniae* IgG antibody titers determined by immunofluorescence test were 1/64. The patient was diagnosed with severe hemolytic anemia complicating *M. pneumoniae* infection. She was treated with clarithromycin (15 mg/kg/d, twice daily) and packed red cell transfusion (10 mL/kg). Then the symptoms were rapidly resolved and hemoglobin increased to 10.4 gr/dL. The antibiotic was continued for a total of 10 days. The patient was discharged in good health after an 8-day hospital stay. In discharge, thrombocyte count, blood chemistry and coagulations studies were normal. Erythrocyte sedimentation rate was 40 mm/hour.

Four months after the discharge, she remained clinically well, her physical examination was normal; her hemoglobin was 13.8 gr/dL; *M. pneumoniae* IgM antibody was negative and *M. pneumoniae* IgG antibody titer was 1/64. Coombs test was found negative.

## 3. Discussion 

We report a case of *M. pneumoniae* infection presenting as severe hemolytic anemia. In this case, pulmonary signs and symptoms were restricted to mild nonproductive cough and interstitial infiltrates on chest radiography. At admission, because of the excessively high erythrocyte sedimentation rate, a hematological malignancy, that is, leukemia, was considered as being the probable diagnosis. But, bone marrow aspiration revealed no changes indicative of a hematological malignancy. In the presence of positive Coombs test, *M. pneumoniae* infection was suspected and diagnosis was subsequently confirmed by the detection of elevated antibody titers to *M. pneumoniae*.

*M. pneumoniae* frequently cause respiratory tract infections, as discussed by Clyde [1]. Most patients with a respiratory infection have a respiratory tract disease without pneumonia and then they have pneumonia. One study suggested that 75–100% of infected patients with *M. pneumoniae* had an intractable, nonproductive cough, while only 3–10% developed pneumonia, as discussed by Mansel et al. [4]. Our patient had nonproductive cough for ten-days, but there were no auscultation findings or dyspnea.

Mycoplasma pneumoniaeinfection has been associated with a variety of extrapulmonary sites. Extrapulmonary manifestations are seen in 20–25% of infected persons and may present before, during, after, or in the absence of pulmonary signs, as discussed by Walts et al. [3]. Extrapulmonary manifestations include hematologic, dermatologic, neurologic, musculoskeletal, renal, cardiac, and gastrointestinal disorders. Hemolytic anemia is the most common hematologic manifestation, and thrombocytopenia, thrombotic thrombocytopenic purpura, hemophagocytosis, and hypercoagulability have been identified as possible hematological complications of *M. pneumoniae* infection, as discussed by Gursel et al. [5]. Subclinical evidence of hemolytic anemia is present in most patients with pneumonia due to *M. pneumoniae*. However, severe hemolysis is extremely rare and is usually associated with severe pulmonary involvement, as discussed elsewhere [6, 7]. Although our patient did not have severe pulmonary disease, she developed severe hemolytic anemia which required blood transfusion. In this respect, our reported case is especially remarkable. 

Antibodies (IgM) against the I antigen on human erythrocyte membranes appear during the course of *M. pneumoniae* infection and produce a cold agglutinin response, as discussed by Daxböck et al. [6]. Serum cold agglutinin titers are elevated in half of adult patients with *M. pneumoniae* infection. However, the formation of cold agglutinins is a nonspecific early IgM reaction against the human erythrocyte I antigen. In children, the accuracy of the cold agglutinin test in detecting *M. pneumoniae* is not known, and the specificity and sensitivity are low, as discussed by Bradley et al. [8]. In our patient, cold agglutinins were also negative, but Coombs test was found positive. A transient elevation in the thrombocyte count was also determined.

Hemolytic anemia associated with *M. pneumoniae* is usually self-limited and resolves spontaneously, but it may require transfusional support, as discussed by Gertz [9]. In autoimmune hemolytic anemia, packed red blood cell transfusions can potentiate hemolysis and their use should be limited, as discussed by Gehrs and Friedberg [10]. Nevertheless, transfusions are indicated and may be lifesaving in patients with severe anemia and/or cardiorespiratory compromise. The risk of transfusion-related hemolysis may be reduced by using an in-line blood warmer at 37°C and keeping the patient warm, as discussed by Gertz [9] Gehrs and Friedberg [10]. Our patient with severe anemia was also transfused one unit of packed red blood cells and was treated with clarithromycin. There is limited, data which suggest that antibiotics are beneficial in mycoplasma associated hemolytic anemia, as discussed by Daxböck et al. [6]. Antibiotics would not be expected to have a major therapeutic role in this setting because hemolytic process is thought to arise from immune-mediated mechanisms. However, treatment of the underlying mycoplasma infection may be associated with more rapid recovery of the hemolytic disease, as discussed by Gertz [9]. Our patient rapidly recovered too after red blood cell transfusion and clarithromycin treatment.

In conclusion, this case demonstrates that severe hemolytic anemia caused by *M. pneumoniae* is not always associated with severe pulmonary involvement. Even in the absence of marked clinical evidence of pneumonia, *M. pneumoniae* may be the cause of severe anemia.
